# Genome Sequences of Seven *Mycoplasma hyosynoviae* Strains Isolated from the Joint Tissue of Infected Swine (*Sus scrofa*)

**DOI:** 10.1128/genomeA.00552-14

**Published:** 2014-06-05

**Authors:** Eric Bumgardner, Russell F. Bey, Weerayuth Kittichotirat, Roger E. Bumgarner, Paulraj K. Lawrence

**Affiliations:** a Newport Laboratories Inc., Worthington, Minnesota, USA; b Systems Biology and Bioinformatics Research Group, Pilot Plant, Development and Training Institute, King Mongkut’s University of Technology Thonburi, Bangkhuntien, Bangkok, Thailand; c Department of Microbiology, University of Washington, Seattle, Washington, USA

## Abstract

*Mycoplasma hyosynoviae* is a Gram-negative bacterium that can cause debilitating arthritis in swine. Currently, there are no *M. hyosynoviae* genome sequences in the GenBank database, which makes it impossible to understand its pathogenesis, nutrition, or colonization characteristics, or to devise an effective strategy for its control. Here, we report the genome sequences of seven strains of *M. hyosynoviae*. Within each genome, several virulence factors were identified that may prove important in the pathogenesis of *M. hyosynoviae*-mediated arthritis and serve as potential virulence markers that may be critical in vaccine development.

## GENOME ANNOUNCEMENT

*Mycoplasma hyosynoviae* is a Gram-negative bacterial parasite that causes arthritis in infected swine. Under normal conditions, the bacterium colonizes the tonsils of infected pigs and remains in this location as a commensal occupant of the nasopharyngeal tract (1). However, the bacterium can also spread systemically through the bloodstream and attach to sites in joint tissue where it can cause lameness and/or clinical arthritis (2). The organism appears to have a global presence (3–5). A recent study has shown that arthritis attributed to *M. hyosynoviae* infection has been increasing in the United States in recent years (1). Despite the importance of this organism as a disease-causing agent in swine, none of the *M. hyosynoviae* genome has been fully sequenced. Here, we report the sequencing of the genomes of seven strains of *M. hyosynoviae* isolated from joint tissues of clinically infected pigs at different locations within North America.

The genome sequences were determined by Illumina sequencing using 250-bp paired-end reads. The reads were assembled into contigs using the *de novo* assemblers Velvet (6) and SPAdes (7) on the Orione instance of Galaxy (8). The contigs from the assemblies were merged using CISA (9), resulting in a final set of six to 10 total contigs in each strain, with *N*_50_ values ranging from 129,749 to 481,749 bp. The total size of the draft genome determined for each strain ranged from 858,952 bp to 936,147 bp. Genome coverage was estimated to range from 38 to 133×. The contigs were aligned against the genome of *Mycoplasma arthritidis* strain 158L3-1 (accession no. CP001047) in Mauve (10). *M. arthritidis* was used because it was the closest available match based on a BLAST search using the largest contig from each strain. Each strain contained two rRNA sequences and a variable number of tRNA sequences, ranging from 25 to 41. At approximately 27% for each genome, the G+C content is typical for a *Mycoplasma* species. The annotation resulted in a total number of coding sequences ranging from 672 bp to 752 bp, of which slightly less than half were able to be functionally annotated. In comparison, *M. arthritidis* 158L3-1 has a genome size of 820,453 bp, with a G+C content of 31%, 3 rRNAs, 32 tRNAs, and 631 annotated genes.

All seven strains presented here encode a complete Opp operon consisting of the *oppA*, *oppB*, *oppC*, *oppD*, and *oppF* genes. A BLAST search using the region of the genome corresponding to the genes in this operon revealed only limited homologies to the corresponding genes of other *Mycoplasma* species. The closest match within the operon is between the *oppB* and *oppC* genes to their corresponding homologs in *Mycoplasma crocodyli* strain MP145 (accession no. CP001991.1). The Opp proteins function primarily as an ATP and oligopeptide transport system that brings nutrients from the host environment into the cell (11). However, the OppA protein has also been shown to function as a means of adhesion to cellular surfaces (11). This operon may encode a primary virulence factor for *M. hyosynoviae*, and the immune response mounted against it may prove important in the defense against infection.

### Nucleotide sequence accession numbers.

The whole-genome shotgun project for *M. hyosynoviae* strains NPL1 to NPL7 has been deposited in the DDBJ/EMBL/GenBank under the following accession no.: NPL1, JFKL00000000; NPL2, JFKK00000000; NPL3, JFKJ00000000; NPL4, JFKI00000000; NPL5, JFKM00000000; NPL6, JFKH00000000; NPL7, JFKG00000000. The versions described here are the first versions, JFKL01000000, JFKK01000000, JFKJ01000000, JFKI01000000, JFKM01000000, JFKH01000000, and JFKG01000000, respectively.
